# Protein Energy Wasting in a Cohort of Maintenance Hemodialysis Patients in Dhaka, Bangladesh

**DOI:** 10.3390/nu14071469

**Published:** 2022-04-01

**Authors:** Tanjina Rahman, Ban-Hock Khor, Sharmela Sahathevan, Deepinder Kaur, Eno Latifi, Mousume Afroz, Esrat Jahan Mitali, Bayan Tashkandi, Nura Afza Salma Begum, Tasnuva Sarah Kashem, Shakib Uz Zaman Arefin, Zulfitri Azuan Mat Daud, Tilakavati Karupaiah, Harun Ur Rashid, Pramod Khosla

**Affiliations:** 1 Department of Nutrition and Food Science, Wayne State University, Detroit, MI 48202, USA; fx7617@wayne.edu (T.R.); kdeepinder@wayne.edu (D.K.); eno.latifi@wayne.edu (E.L.); bayan.tashkandi@wayne.edu (B.T.); 2 Institute of Nutrition and Food Science, University of Dhaka, Dhaka 1000, Bangladesh; 3 Faculty of Food Sciences and Nutrition, Universiti Malaysia Sabah, Kota Kinbalu 56000, Malaysia; khorbanhock@gmail.com; 4 Department of Allied Health Sciences, Universiti Tunku Abdul Rahman, Kampar 31900, Malaysia; sham_0901@yahoo.com; 5 Department of Nephrology, Kidney Foundation Hospital and Research Institute, Mirpur, Dhaka 1216, Bangladesh; mafroz.sumona@gmail.com (M.A.); esrat.wasi@gmail.com (E.J.M.); nuraafzanupur@yahoo.com (N.A.S.B.); tasnuva.kashem@gmail.com (T.S.K.); shakib04@yahoo.com (S.U.Z.A.); rashid@bol-online.com (H.U.R.); 6 Department of Dietetics, Faculty Medicine and Health Sciences, Universiti Putra Malaysia, Serdang 43400, Malaysia; zulfitri@upm.edu.my; 7 School of Medicine, Faculty of Health Sciences, Taylors University, Subang Jaya 47500, Malaysia; tilly_karu@yahoo.co.uk

**Keywords:** dyslipidemia, protein energy wasting, Bangladeshi hemodialysis patients, nutrition status

## Abstract

Malnutrition is associated with high rates of mortality among patients with end stage kidney disease (ESKD). There is a paucity of data from Bangladesh, where around 35,000–40,000 people reach ESKD annually. We assessed protein-energy wasting (PEW) amongst 133 patients at a single hemodialysis setting in Dhaka. Patients were 49% male, age 50 ± 13 years, 62% were on twice-weekly hemodialysis. Anthropometric, biochemical, and laboratory evaluations revealed: BMI 24.1 ± 5.2 kg/m^2^, mid-arm muscle circumference (MAMC) 21.6 ± 3.6 cm, and serum albumin 3.7 ± 0.6 g/dL. Based on published criteria, 18% patients had PEW and for these patients, BMI (19.8 ± 2.4 vs. 25.2 ± 5.2 kg/m^2^), MAMC (19.4 ± 2.4 vs. 22.2 ± 3.8 cm), serum albumin (3.5 ± 0.7 vs. 3.8 ± 0.5 g/dL), and total cholesterol (135 ± 34 vs. 159 ± 40 mg/dL), were significantly lower as compared to non-PEW patients, while hand grip strength was similar (19.5 ± 7.6 vs. 19.7 ± 7.3 kg). Inflammatory C-reactive protein levels tended to be higher in the PEW group (20.0 ± 34.8 vs. 10.0 ± 13.9 *p* = 0.065). Lipoprotein analyses revealed PEW patients had significantly lower low density lipoprotein cholesterol (71 ± 29 vs. 88 ± 31 mg/dL, *p* < 0.05) and plasma triglyceride (132 ± 51 vs. 189 ± 103 mg/dL, *p* < 0.05), while high density lipoprotein cholesterol was similar. Nutritional assessments using a single 24 h recall were possible from 115 of the patients, but only 66 of these were acceptable reporters. Amongst these, while no major differences were noted between PEW and non-PEW patients, the majority of patients did not meet dietary recommendations for energy, protein, fiber, and several micronutrients (in some cases intakes were 60–90% below recommendations). Malnutrition Inflammation Scores were significantly higher in PEW patients (7.6 ± 3.1 vs. 5.3 ± 2.7 *p* < 0.004). No discernible differences were apparent in measured parameters between patients on twice- vs. thrice-weekly dialysis. Data from a larger cohort are needed prior to establishing patient-management guidelines for PEW in this population.

## 1. Introduction

Chronic kidney disease (CKD) is a major global public health problem, and this is exacerbated in individuals who progress to end stage kidney disease (ESKD), requiring renal replacement therapy (RRT)—either dialysis or a transplant. In a community survey of an urban based population in Bangladesh, 26% of adults met the criteria for CKD, of which 62% were Stages 1 and 2 [1]. Data from hospital, urban, and underprivileged populations suggest a CKD prevalence of 16% to 18% in Bangladesh; of them 11% are stage 3 and above [2]. Annually, around 35,000–40,000 Bangladeshis reach ESKD. The existing facilities can hardly accommodate 9000–10,000 new patients, which means, RRT is not available for almost two-thirds of ESKD patients and consequently, about 40,000 people die each year from kidney disease [2]. For the small number of patients who can access hemodialysis (HD), outcomes are far from ideal with 1-, 3-, and 5-year survival rates of 95%, 80%, and 55%, respectively [2]. Patients with CKD and ESKD also have a higher risk of developing cardiovascular disease (CVD), associated with inflammation, malnutrition, stroke, dyslipidemia, a compromised antioxidant system, metabolic abnormalities, and early death [3]. However, in Bangladesh, there is a greater focus on population control, provision of clean drinking water, and eradication of communicable diseases [4], while the focus on management of CKD is sub-optimal.

The syndrome of protein-energy wasting (PEW), a multi-factorial, maladaptive metabolic state, characterized by a loss of body protein mass and energy reserves is a major cause of morbidity and mortality amongst patients with CKD and ESKD [5]. Unlike malnutrition (due to inadequate nutrient intake), PEW is common in an inflammatory environment and generally resistant to nutrition supplementation. Global prevalence of PEW, based on a meta-analysis of 90 studies from 34 countries including 16,434 patients, was estimated to be between 11–54% in patients with CKD Stages 3–5 and between 28–54% in dialysis patients [5]. Additionally, quality of life is significantly impacted by PEW. In a cohort of 331 maintenance hemodialysis (MHD) patients, a positive association was found between prolonged poor appetite, inflammation, and quality of life (QoL) [6]. The nutritional status of HD patients in Bangladesh is poorly documented and no information is available on the prevalence of PEW. This may, in part, be attributed to a lack of appropriate renal nutrition knowledge of personnel employed in the dialysis centers. A recent survey from 155 countries revealed that 52% of these do not employ renal dietitians/nutritionists to provide nutrition counseling, which is the case in Bangladesh [7]. 

In most developed countries, frequency of HD is typically thrice-weekly as costs are generally covered by health-insurance providers. However, in developing countries [8], poor socioeconomic status, a paucity of facilities, and insufficient governmental financial support results in diminished weekly dialysis frequency (twice-weekly). Exceptions include Mexico, where weekly dialysis treatment averages 1.2 sessions [9], while in Guatemala it can be once to thrice-weekly depending on the insurance provider [10]. Costs for dialysis, as well as any blood tests or recommended oral nutrition supplements, are frequently borne by patients in developing countries. Poor survival outcomes in Bangladeshi HD patients may be associated with the twice-weekly dialysis regimen. In Lithuania, a study with 2063 MHD patients demonstrated that patients on once- and twice-weekly therapy had a two-fold higher risk for mortality compared to those on thrice-weekly dialysis [11]. However, in a subgroup analysis of the HEMO study, women experienced a survival benefit with higher dialysis dose, whereas men did not [12]. Additionally, there is now a renewed interest as to whether instant transition to dialysis should be replaced by a gradual transition. Therefore, data on the effectiveness of thrice-weekly dialysis versus twice-weekly (or less frequent dialysis), and their association with patients’ nutritional status, is lacking.

Advanced CKD patients, as well as those on MHD, are at higher risk for CVD, partly attributed to a dyslipidemic state characterized by high triglyceride (TG) and low plasma high density lipoprotein cholesterol (HDL-C) [13], which can be used collectively to predict arterial stiffness and cardiovascular events in even apparently healthy adults [14]. It is also a determinant of poor glucose homeostasis, glycemic control, and microangiopathy [15]. 

Accordingly, the objective of the current study was to document nutritional, biochemical, as well as other health-related parameters in MHD patients at the Kidney Foundation Hospital and Research Institute (KFHRI) in Dhaka, Bangladesh, where >360,000 dialysis sessions have been provided for patients with kidney failure over the last 16 years. We explored prevalence of PEW (as the recent meta-analysis of global PEW did not have any data for Bangladesh [16]), evaluated nutritional and health parameters, and also assessed lipoproteins. Finally, we compared selected parameters between patients undergoing twice- versus thrice-weekly dialysis to observe whether there is any association between dialysis frequency and patients’ health outcomes. 

## 2. Materials and Methods

### 2.1. Patients and Study Design

The study was approved by the ethics boards of KFHRI and Wayne State University (IRB #123314M1F). Patients undergoing MHD were assessed following identification by local hospital staff and inclusion criteria were ESKD patients undergoing MHD treatment for at least 3 months and >18 years old. Patients with hepatitis were excluded. Informed written consent was obtained from all patients. Where needed, consent forms and case report forms were translated into the Bangla language, approved by bi-lingual nephrologists and a local registered lawyer. All procedures and methods were in accordance with institutional approvals. A total of 133 patients were enrolled (Supplementary Table S1). Of these, 102 were participants in a clinical trial assessing the impact of supplementation with 300 mg of tocotrienols or placebo (PATCH clinical trial NCT 02358967). The data reported in the current study from the “PATCH participants” were collected prior to the start of their supplementation.

### 2.2. Anthropometric and Nutritional Assessments

To facilitate anthropometric and nutrition data collection, three rounds of training were implemented. Initially a training workshop was conducted at KFHRI for nurses, medical doctors, and the local nutritionists. (There is no formal dietetics program or specialized nutrition program with practicums in renal nutrition in Bangladesh and most nutritionists are individuals who took some nutrition courses as undergraduates. Hence the use of the term “nutritionist” in Bangladesh will have different connotations as compared to, e.g., “nutritionists” in Europe or the USA). Subsequently, one member affiliated with the hospital site attended a two-week training workshop in Malaysia with other members of the research team. During this visit, in addition to formal lectures as well as practicums in anthropometric assessments, shadowing of staff in local dialysis units was arranged. A final training session at KFHRI was conducted by members of the research team, just prior to data collection, again involving nurses, medical doctors. and resident nutritionists. Collectively in these sessions, training was provided by research team members who were registered dietitians (BHK, SS, ZAMD, and TK accreditation from Malaysia, Australia, and the USA), as well as an individual (TK) who was Level 3 certified for anthropometric assessments by the International Society for Anthropometry and Kinanthropometry (ISAK).

Anthropometric assessments included measurement of body mass index (BMI) from height and post-dialysis weight [17]. Mid-arm circumference (MAC) was measured for each subject in a standing position using a non-stretchable Lufkin^®^ tape (Apex Tool Group, LLC, Apex, NC, USA). Triceps skinfold thickness (TSF) was measured using Lange skinfold calipers (Lange Skinfold Calipers, Power System, Knoxville, TN, USA). Handgrip strength for each patient was measured by taking three readings, with a rest period of at least 1 min between the trials from the non-fistula hand, using a Jamar Hand-grip dynamometer (BK-7498; Fred Sammons, Inc., Burr Ridge, IL, USA) following the standard protocol from the American Society of Hand Therapists [18].

A 24-h diet recall was collected for a non-dialysis day for each patient using household measures. Additionally, pictures and weights of common food items were taken to determine the amount of food consumed by patients using cooked food provided by the hospital canteen and in local households. Dietary data were then analyzed using the *ESHA* Food Processor Nutrition Analysis and Fitness Software, version 11.3.285. For mixed dishes, which were not in the database, ingredients were entered in the recipe builder within the software. Approximately 150 Bangladeshi recipes were constructed based on the “Food Composition Table for Bangladesh” [19] and information available online. To minimize systemic error, underreporting of dietary data was evaluated by calculating the ratio between reported energy intake (EI) and basal metabolic rate (BMR). Goldberg cut-off equations for EI: BMR (Energy Intake: Basal metabolic rate) were used to determine under-reporters, with a ratio of EI:BMR < 0.75 used as a cut-off for under-reporters, while a ratio >2.4 was considered for over-reporters [20]. BMR was calculated using the Harris–Benedict Equation. When weight was <95% or >115% of the standard body weight, adjusted edema-free body weight was used [21], otherwise actual body weight as recommended by NKF KDOQI (2000) guidelines was used [22].

Sociodemographic data were collected from patient’s medical records. Health related questionnaires, such as malnutrition inflammation score (MIS), appetite and diet analysis tool (ADAT), and health-related Quality of life (HR-QoL), were also administered. MIS is a useful tool that can help identify MHD patients, who are susceptible to premature death. A score of more than 4 to 5 indicates high risk of patients’ mortality [23] (see Table S1: Study Flow Chart).

### 2.3. Diagnosis of PEW Patients

PEW was characterized in the patients based on anthropometric and biochemical measures as detailed by the International Society for Renal Nutrition and Metabolism (ISRNM) [5]. These utilize data from four criteria with pre-established values—*serum chemistry* (albumin, pre-albumin or cholesterol), *body mass* (BMI, unintentional weight loss over time or fat percentage), *muscle mass* (muscle wasting, MAMC or creatinine appearance), and *dietary intake* (using measures of dietary protein intake (DPI) or dietary energy intake (DEI). An individual was classified as having PEW if measures for three out of four major criteria were met.

### 2.4. Other Nutrition and Health Status Assessment 

An ADAT to evaluate appetite and factors affecting dietary intake was administered [24]. A MIS was calculated to assess the severity of malnutrition-inflammation complex syndrome [25]. This is a combination of seven components of subjective global assessment along with BMI, serum albumin, and total iron binding capacity (TIBC). The cumulative score for MIS ranges between 0 (*normal*) to 30 (*severely malnourished*). A measure of health-related quality of life (HRQOL), Kidney Disease Quality of Life 36-item surveys (KDQOL-36), was also administered [26]. The KDQOL-36 comprises five subscales calculated separately: (1) SF-12 physical component summary (PCS), (2) SF-12 mental component summary (MCS), (3) burden of kidney disease, (4) symptoms of kidney disease, and (5) effects of kidney disease. Subscale scores range from 0 to 100, with lower scores indicating poor self-reported QoL [24,27].

### 2.5. Blood Sampling and Lipid Measurements

Non-fasting blood samples were collected into two sets of tubes, one containing Ethylenediaminetetraacetic acid and one containing Lithium Heparin (10 mL each). The use of non-fasting samples was in line with recent reports on their validity for assessing CVD risk, which is consistent with recent guidelines [28]. Plasma samples were isolated on site at the hospital by centrifugation at 3500 rpm for 10 min at 4 °C and multiple aliquots were immediately stored at −80 °C.

Plasma samples (on dry ice) were air-shipped to Michigan (World Courier Service, Bangladesh). Plasma total cholesterol (TC) and TG were measured using enzymatic assays (Pointe Scientific Inc., Canton, MI, USA). HDL-C was assessed in the supernatant after precipitating apo B containing lipoproteins (Point Scientific Inc.) as detailed by the manufacturer’s protocol. Low density lipoprotein cholesterol (LDL-C) was calculated using the Friedwald equation: [LDL cholesterol = Total cholesterol − HDL cholesterol-(TG/5)]. HDL and LDL, were analyzed in plasma using the Lipoprint^TM^ polyacrylamide electrophoresis-based system (Quantimetrix Corporation, Redondo Beach, CA, USA) as per the manufacturer’s protocol and data quantitated using the associated software as detailed previously [29]. The Lipoprint^TM^ system is U.S. Food and Drug Administration (FDA) certified for LDL measurements, while values for HDL are for research purposes only.

### 2.6. Statistical Analyses

Data were analyzed using SPSS version 24 (IBM, SPSS Statistics Inc., Chicago, IL, USA). Descriptive statistics of continuous variables are presented as means ± standard deviation, median (interquartile range, IQR) or frequency (percentage). The normal distribution of continuous variables was assessed using a Kolmogorov–Smirnov test. Differences between groups were analyzed using Student *t* test, one-way ANOVA, and Mann–Whitney’s U test for normally distributed and non-normally distributed data, respectively. *p* < 0.05 was considered significant.

## 3. Results

Assessments were carried out in 133 patients (Table 1) of which 49% were males. Mean age was 50 ± 13 years, average duration of a dialysis session was 3.8 ± 0.4 h, mean dialysis vintage 30.0 ± 24.3 months and 62% patients underwent twice-weekly dialysis. Causes of developing ESKD were hypertension (HTN) 39%, followed by diabetic nephropathy (DN) 28%, and chronic glomerulonephritis (CGN) 18%. Other reasons (8%) reported by the patients were: adult polycystic kidney disease (ADPKD), kidney stones, postpartum complications of acute kidney injury complicated to chronic kidney disease, or unknown reasons.

Data to assess for PEW, (based on ISRNM criteria) were available from 116 of the 133 patients (87%). In these 116 patients, 58 (49%) had BMI < 23 kg/m^2^ while 75 (57%) had MAMC values 10% below the 50th percentile of reference. Total cholesterol values were below 100 mg/dL in only six patients (5%). Albumin levels, available for 105 of these patients revealed 64 (61%) patients with values < 3.8 g/L. Collectively, as a result of satisfying three of the four ISRNM criteria, 20 patients (17.4%) were classified as having PEW, while 95 patients were designated Non-PEW (Table 1). Within the two groups, 67% of the PEW patients and 43% of Non-PEW were males. Age, duration of dialysis, dialysis vintage, and dialysis frequency were similar in both groups. Causes of ESRD were HTN 35% vs. 44%, 20% vs. 28%, CGN 35% vs. 13%, and other 5% vs. 13% in PEW vs. Non-PEW, respectively (Table 1). Consistent with the ISRNM criteria, PEW patients had significantly lower BMI (19.8 ± 2.4 vs. 25.2 ± 5.2 kg/m^2^, *p* < 0.001), MAC (22.4 ± 2.4 vs. 27.5 ± 5.2 cm, *p* < 0.001) MAMC (19.4 ± 2.4 vs. 22.2 ± 3.8 cm, *p* < 0.05), and serum albumin (3.5 ± 0.6 vs. 3.8 ± 0.5, g/dL, *p* < 0.05). The lower BMI was attributed to lower dry weight (50.7 ± 9.1 vs. 62.7 ± 12.3 kg, *p* < 0.03). PEW patients also had significantly lower TSF (9.6 ± 3.7 vs. 17.2 ± 8.2 mm, *p* < 0.001). No significant differences were noted in HGS, TIBC, URR%, serum Na, K, P, ferritin, or Kt/V.

Clinical data measured at the hospital indicated minor differences in patients undergoing twice- vs. thrice-weekly dialysis. Observed differences in select measured parameters between patients on twice- vs. thrice-weekly dialysis included dialysis vintage and ferritin levels. (Supplementary Table S2). Thus, any significant associations between dialysis frequency and nutrition and health outcomes of MHD patients were not discernible in this study.

Analyses of diet data from the 115 patients assessed for PEW revealed 66 to be acceptable reporters (AR). Collectively, for these 66 patients, macronutrient analyses (Table 2) revealed a DEI of 24.2 ± 7.6 kcal/Kg BW/d vs. a KDOQI recommendation of 30–35 kcals/Kg BW/d. The dietary protein intake of 0.9 ± 0.3 g/Kg BW/d was also less than the recommended values of 1.2 g/Kg/BW/d. While phosphorus intake of 14.2 ± 5.4 mg/Kg BW/d was within recommended levels (10–17 mg/Kg BW/d), the phosphorus/protein ratio of 16.5 ± 3.9 mg/g protein was higher than recommended values of <12 mg/g protein. Amongst the micronutrients, the patients consumed substantially lower amounts of Vitamin D (46 ± 39 IU), E (2.3 ± 1.6 mg), K (22 ± 78 μg), biotin (10.0 ± 9.8 μg), and folate (142 ± 189 μg) as compared to KDOQI recommendations of 600 IU, 90–120 μg, 30 μg, and 1000 μg, respectively (Table 2). Intakes of dietary calcium (431 ± 262 mg), phosphorus (872 ± 422 mg), sodium (2099 ± 918 mg), zinc (8.0 ± 4.5 mg), and magnesium (231 ± 91 mg) were in line with KDQOI recommendations. Comparisons between PEW and Non-PEW groups, solely amongst the AR, revealed significant differences only in calcium and polyunsaturated fatty acid intakes. In terms of macronutrient distribution, carbohydrates represented ~58% of total calories, while dietary fat accounted for ~25–27%. The distribution of macronutrients was similar between PEW and Non-PEW patients (data not shown).

Various questionnaires were used in the present study to assess the patients’ health and nutritional status. A statistically significant and higher malnutrition inflammation score (MIS) of 7.6 was found among PEW patients compared to a lower score of 5.3 in their non-PEW counterparts. The mean ADAT score, KDQoL, and burden of kidney disease were not significantly different between the PEW and Non-PEW groups (Table 3).

Plasma lipids and lipoprotein subfractions for the PEW and Non-PEW patients are shown in Table 4. PEW patients had significantly lower TC, TAG, and LDL-C as compared to Non-PEW patients. Ratios of TC/HDL-C, LDL-C/HDL-C, and TAG/LDL-C all were significantly lower among PEW patients. LDL particles diameters were significantly higher in PEW patients. PEW patients had significantly lower cholesterol in small sized LDL particles and significantly more cholesterol in large-sized HDL particles and less cholesterol in small-sized HDL particles. Analyses of lipoprotein particle sizes revealed significantly smaller LDL particles 267 ± 7 vs. 271 ± 3 Angstroms, *p* < 0.005) in the Non-PEW group.

## 4. Discussion

In the present study, we evaluated demographics, anthropometric, dietary, biochemical, and other laboratory parameters in a group of patients on MHD in a specialized kidney hospital in Bangladesh. To the best of our knowledge, this is one of the first multiple data sets for MHD patients from Bangladesh that has evaluated parameters that are routinely measured in other countries, to reflect nutritional status. As such, our data will serve as a useful reference for future work. Major findings from our study warrant attention. *First*, the vast majority of patients failed to meet KDQoL guidelines for energy, protein, and numerous micronutrients [30]. The failure to meet micronutrient requirements is important give their varied roles impacting protein metabolism. Numerous enzymes impacted by vitamin co-factors, can influence protein expression, by virtue of their ability to act as antioxidants, nuclear receptor agonists, and signal transducers [31] *Second*, the prevalence of PEW among patients on MHD in KFHRI was estimated at 18% (24% and 11% among males and females, respectively). Prior to our study, PEW amongst HD patients in Bangladesh has not been given importance in terms of being a health issue. We believe that PEW prevalence may be even higher since blood chemistry data (e.g., serum albumin, TC), diet assessments (energy and protein intakes), and anthropometric data (BMI, MAMC)—critical components of PEW assessment based on ISRNM criteria—are not routinely carried our across dialysis facilities in Bangladesh. This is primarily because patients typically bear the costs for blood work, while the dialysis facilities have a paucity of trained personnel that can carry out diet and anthropometric assessments. While data are needed to establish the prevalence of PEW in a much larger cohort, a coordinated effort also needs to be undertaken to evaluate certain basic parameters across all dialysis facilities.

A recent study in Bangladesh [32], which was conducted in a rural clinical setting, stated that 57% of their study population had a BMI less than 23 kg/m^2^, which was similar to our data. In the current study population, 49% had a BMI less than 23 kg/m^2^. Additionally, 57% patients showed lower MAMC compared to the values that are 10% below the 50th percentile reference population and 61% had a serum albumin of <3.8 g/dL. A similar trend was found in a recent study conducted in Indonesia, where 66% of patients had a lower MAMC and serum albumin of <3.8 g/dL [33]. Another robust anthropometric tool that was used in our study was the measurement of HGS. Currently, there are no standardized tables of HGS for MHD population. The reference HGS value in kg for a 55-year-old right-handed male is 21.7 [34,35], which is almost certainly not appropriate for dialysis patients. In our study population, the mean age was 50 years and 19 Kg, which is ~10% lower as compared to what was found among healthy adult population. The value was also similar to HGS reported in HD patients from Saudi Arabia [36] and Malaysia [37] of comparable age.

Serum transferrin could be measured by using serum TIBC (total iron binding capacity) and is considered as an acceptable marker of malnutrition in patients on MHD. One study showed that MHD patients with a serum TIBC of >250 mg/dL had a high BMI and low serum ferritin level, and thus a low risk of inflammation and death compared to patients with a low TIBC level [38]. In our study, the mean serum TIBC was 242 ± 64 mg/dL and it was lower among PEW patients. Studies have shown that, high serum ferritin level of more than 250 ng/mL are associated with high risk of mortality among patients with CKD [39].

Mean ferritin levels of our study patients (Table 2) were 497 ± 443 ng/mL (646 ± 543 ng/mL) in the PEW patients), while 18% of patients had a ferritin of >2000 ng/mL. Despite the variation, both findings indicate an increased mortality risk in this patient pool. In addition, we assessed C-reactive protein (CRP) as an inflammatory marker and found that, mean CRP levels of our study population were 14.5 ± 25.8 mg/L (higher for PEW patients), another predictor of increased cardiovascular and mortality risk in this patient pool [40].

No significant differences were observed in other components of health-related questionnaires, (which might be due to the small number of PEW patients) with the exception of MIS score. However, an MIS score of ≥5 is considered as an indicator of the prevalence of malnutrition in many studies and from this point of view, we can further predict that, the number of PEW patients identified in this study might be an underestimate [23,41].

Estimating dietary intake is challenging in patients with chronic diseases [40,42]. In this study, we collected one-day 24-h diet recalls and nutrient analyses was possible from 61% of these (acceptable reporters). About 39% of our study patients were under-reporters, which is a common scenario among MHD patients, especially women and those with a high BMI [43]. Amongst all patients (PEW and non-PEW) protein intakes of 54 ± 21 g/d and energy intakes of 1429 ± 497 kcal/d were noted. While we did not assess diet intake in normal non-dialysis patients in this study, previous data from a normal urban population revealed protein intake estimates of 68–78 g/day, with energy intake estimates of 2142 to 2394 kcal/d [44]. However, this was the first analysis of diet data in Bangladesh using an internationally accepted software and additional effort was given to improve the quality of data.

The TG/HDL ratios observed in this study are considerably higher than our previous reports in HD patients from Malaysia, Saudi Arabia, as well as a cohort of African American HD patients in the USA [36,37,45]. As ours is a preliminary observational study, the underlying reason for this is not clear. While South Asians in general have lower HDL levels due to genetics, differences in diet quality, specifically macronutrient intake may also be partly responsible. The current cohort consumed a larger proportion of calories from carbohydrates, which has been shown to contribute to the different dietary patterns in Malaysian HD patients [37] as well as AA HD patients [45]. Future studies with a larger number of acceptable reporters will help delineate possible dietary contributors to the circulating lipid profiles. The small atherogenic LDL particle sizes noted in the current study are consistent with previous reports from other populations [36,45].

The presence of a preponderance of small or large HDL particles and their role in contributing to increased mortality in HD patients have been the subject of some debate. While some studies have revealed small HDL particles predominate, others have noted increases in large HDL particles in HD patients [44,45,46,47]. In addition, dysfunctional HDL particles (HDL particles unable to deliver cholesterol to the liver for excretion) may also contribute to increased mortality in HD patients [48,49]. In our study, lack of mortality data precludes any conclusions regarding whether the observed decrease in the proportion of small and intermediate-sized HDL particles (with a corresponding increase in the proportion of large HDL particles) increases morality in the DL patients.

### Limitations and Strengths of Our Study

Several study limitations need to be noted. *First*, data were collected from one hospital in Dhaka, and in all likelihood are not representative of the country. *Second*, only one 24 h diet recall was captured, limitations of which have been discussed by others. *Third*, home-cooking involved numerous recipes, all of which may not have been captured by the patients. *Fourth*, while Bangladesh Food composition tables were used, these are not exhaustive, and no software captures this information. For this reason, local food items were incorporated manually into the software for the first time to obtain a better picture of patients’ dietary intake. *Fifth*, PEW may have been underestimated as data were not available for all patients for the measures required for PEW assessment. With reference to the latter, it should be pointed out that in several Western countries (e.g., USA) where dialysis has been available with almost universal coverage since the early 1980s, the direct cost to the patient is minimal. In contrast, in other parts of the world (e.g., Bangladesh) dialysis costs are borne by the patients. Additionally, costs for various blood tests (e.g., albumin, measurement of lipids) have to be met by the individual and such data are not always available. Hence specific tests need to be availed based on patient needs and economic status. A uniform electronic database to capture historical blood parameters is not available. As such, gathering robust and comprehensive data sets for research purposes was challenging. Our initial experience suggests that training personnel in targeted measures (e.g., anthropometric and diet data collection and analyses) which involve no costs to patients can be an important contributor in filling this gap. It should be noted that there is no formal dietetics program or practicum nutrition training in Bangladesh. Additionally, based on our analyses, we speculate that if all HD clinics in Bangladesh were to do mandatory measurements of serum albumin along with standardized anthropometric assessments, this may help capture a truer picture of PEW, and facilitate its assessment and subsequent management. Once such data are available, they would facilitate appropriate nutrition recommendations and interventions for HD patients.

Our study has several strengths. First, we were able to capture anthropometric, dietary, and biochemical parameters from the same set of patients, which to our knowledge is one of the first from dialysis patients in Bangladesh. Second, a coordinated training effort across Malaysia, Bangladesh, and the USA ensured uniform protocols for diet and anthropometric assessment. The latter were modeled on the land marking of patients established by ISAK for anthropometric assessments. Third, the presence of overseas personnel to supervise and train local staff in data collection helped ease logistics. Fourth, the fact that the hospital had patients whose standard care included twice- or thrice-weekly dialysis allowed us to capture some initial data on individuals receiving twice-/thrice-weekly dialysis. Finally, our lipid analyses were one of the first to provide information on individual lipoproteins (as opposed to total cholesterol) in this population. It will be important in future studies to assess lipids in pre-dialysis CKD Stage 3–4 patients to assess if aggressive lipid management is warranted.

## 5. Conclusions

In this initial report we assessed nutritional, anthropometric, and lipid parameters in MHD patients in a single center in Bangladesh. Our data revealed suboptimal intakes for numerous micronutrients, protein, and energy, and just under 20% of the patients had PEW. Plasma lipoprotein analyses revealed dyslipidemia, characterized by elevated TG/HDL-C ratios primarily attributed to low HDL-C concentrations. The long-term implications of these parameters on morbidity and mortality, as well as nutritional management of MHD patients, need to be determined.

## Figures and Tables

**Table 1:** Demographics, anthropometric and biochemical parameters: PEW versus Non-PEW.

Parameters	All	PEW	Non-PEW	*p* Value
**Gender M/F (*n*)**	65/68 (133)	13/7 (20)	41/54 (95)	
**Age (Years)**	49.8 ± 13.0 (133)	48.6 ± 17.5 (20)	49.9 ± 11.5 (95)	0.674
**Duration of dialysis, h**	3.8 ± 0.4 (120)	3.9 ± 0.3 (18)	3.8 ± 0.4 (91)	0.242
**Dialysis vintage, months**	30.0 ± 24.3 (123)	32.1 ± 32.8 (19)	29.0 ± 22.2 (95)	0.621
Dialysis frequency, *n* ** (%)				
***Thrice a week***	49 (38%)	7 (35%)	34 (36%)	
***Twice a week***	81 (62%)	13 (65%)	61 (64%)	
**Causes of ESRD, *n* (%)**				
***HTN***	52 (39%)	7 (35%)	42 (44%)	
***DN***	35 (26%)	4 (20%)	27 (28%)	
***CGN***	23 (17%)	7 (35%)	12 (13%)	
***Others***	13 (10%)	1 (5%)	12 (13%)	
***Unknown***	10 (8%)	1 (5%)	2 (2%)	
**Height (cm)**	158.5 ± 9.3 (116)	159.7 ± 8.3 (20)	158.3 ± 9.6 (95)	0.539
**Dry weight (kg)**	60.6 ± 12.6 (116)	50.7 ± 9.1 ^a^ (20)	62.7 ± 12.3 ^a^ (95)	<0.001
**BMI (kg/m^2^)**	24.2 ± 5.2 (116)	19.8 ± 2.4 ^a^ (20)	25.2 ± 5.2 ^a^ (95)	<0.001
**<23 kg/m^2^**	20.1 ± 2.0 (55)	19.9 ± 2.4(20)	20.3 ± 1.7 (34)	
**≥23 kg/m^2^**	27.9 ± 4.5 (61)	-	27.9 ± 4.5 (61)	
**HGS (kg)**	19.8 ± 7.4 (116)	19.5 ± 7.6 (20)	19.7 ± 7.3 (95)	0.938
**MAC (cm)**	26.6 ± 5.2 (116)	22.4 ± 2.4 ^a^ (20)	27.5 ± 5.2 ^a^ (95)	<0.001
**TSF (mm)**	15.8 ± 8.2 (116)	9.6 ± 3.7 ^a^ (20)	17.2 ± 8.2 ^a^ (95)	<0.001
**MAMC (cm)**	21.7 ± 3.7 (116)	19.4 ± 2.4 ^a^ (20)	22.2 ± 3.8 ^a^ (95)	<0.001
**Reduction > 10%**	19.7 ± 2.9 (65)	19.4 ± 2.4(20)	19.9 ± 3.1 (44)	
**Reduction ≤ 10%**	24.1 ± 3.1(51)	-	24.1 ± 3.1 (51)	
**Albumin (g/dl)**	3.7 ± 0.6 (97)	3.5 ± 0.6 ^a^ (19)	3.8 ± 0.5 ^a^ (77)	0.029
**<3.8 g/dl**	3.4 ± 0.4 (57)	3.4 ± 0.6(18)	3.5 ± 0.3 (38)	
**≥3.8 g/dl**	4.1 ± 0.5(40)	4.7(1)	4.1 ± 0.5 (39)	
**TC (mg/dL)**	162 ± 51 (116)	135 ± 34 ^a^ (20)	159 ± 40 ^a^ (95)	0.01
**<100 mg/dL**	94 ± 4 (4)	95 ± 4 (3)	90 (1)	
**≥100 mg/dL**	164 ± 50 (112)	142 ± 32 (17)	168 ± 52 (94)	0.058
**TIBC (mg/dL)**	244.1 ± 61.5 (81)	228.1 ± 55.4 (15)	247.7 ± 62.6 (66)	0.267
**URR%**	65.3 ± 8.8 (83)	67.8 ± 8.8 (15)	65.0 ± 8.6 (67)	0.231
**Na (mEq/L)**	136.1 ± 3.8 (107)	136.4 ± 2.5 (18)	136.0 ± 4.1 (88)	0.731
**K (mEq/L)**	5.0 ± 0.7 (112)	5.2 ± 0.7 (19)	5.0 ± 0.7 (92)	0.247
**P (mg/dl)**	4.5 ± 2.2 (100)	4.6 ± 2.7 (17)	4.5 ± 2.1 (82)	0.811
**CRP (mg/L)**	14.5 ± 25.8 (95)	20.0 ± 34.8 (17)	10.0 ± 13.9 (69)	0.065
**Ferritin (ng/mL)**	496.7 ± 442.8 (69)	645.8 ± 543.4 (9)	482.0 ± 423.1 (59)	0.302
**F > 2000 ng/mL**	15	6	9	
**Kt/V**	1.3 ± 0.4 (55)	1.4 ± 0.4 (11)	1.3 ± 0.4 (44)	0.435

Values are mean ± SD for the numbers in parentheses. From the pool of 133 patients, relevant data for PEW assessment (based on ISRNM guidelines) were available from 115 patients. (20 were PEW and 95 were Non-PEW patients). Biochemical data were obtained from patient’s medical records. HTN: Hypertension, DN: Diabetic nephropathy, CGN: Chronic glomerulonephritis, Other: adult polycystic kidney disease, kidney stone, unknown, postpartum complication; ESRD: End-stage renal disease. BMI: Body mass index, HGS: Hand grip strength, MAC: Mid-arm circumference, TSF: Triceps skin fold, MAMC: Mid-arm muscle circumference, TIBC: Total iron binding capacity. F: Ferritin. URR%: Urea reduction rate. Na: Sodium, K: Potassium, P: Phosphorous. ^a^ Mean values (between PEW and Non-PEW patients) sharing a common superscript were significantly different from each other using a one-way ANOVA (*p* < 0.05). ** One patient was dialyzed once a week and was not included in any analyses.

**Table 2:** Dietary analysis for acceptable reporters between PEW and Non-PEW groups.

Nutrients	All (*n* = 65)	PEW (*n* = 13)	Non-PEW (*n* = 52)	*p* Value	KDOQI Guidelines
**Calories (Kcal)**	1429 ± 497	1327 ± 278	1455 ± 537	0.412	**
**DEI/Kg BW/day**	24.2 ± 7.6	26.2 ± 5.9	23.7 ± 7.9	0.289	30–35 Kcal/Kg BW/day
**<25**	19.1 ± 3.4 (38)	19.0 ± 2.3 (5)	19.2 ± 3.4 (33)		
**≥25**	31.4 ± 5.8 (27)	30.2 ± 3.1 (8)	32.3 ± 6.8 (19)		
**Protein (g)**	53.6 ± 21.0	55.2 ± 19.5	53.7 ± 22.4	0.852	**
**DPI/Kg BW/day**	0.9 ± 0.3	1.1 ± 0.3 ^a^	0.9 ± 0.3 ^a^	*0.035*	1.0–1.2 g/Kg BW/day
**<1.0**	0.7 ± 0.1 (38)	0.7 ± 0.1 (6)	0.6 ± 0.1 (32)		
**≥1.0–1.2**	1.2 ± 0.3 (27)	1.2 ± 0.3 (7)	1.2 ± 0.2 (20)		
**P mg/Kg BW/day**	14.7 ± 5.6	15.2 ± 3.2	14.5 ± 6.1	0.695	10–17 mg/Kg BW/day
**P/Protein**	16.5 ± 3.9	14.9 ± 3.7	16.9 ± 3.9	0.088	<12 mg/g of protein
**Carbohydrates (g)**	207 ± 71	192 ± 44	211 ± 76	0.401	**
**Total Fiber (g)**	17 ± 7	15 ± 4	17 ± 7	0.298	20–25 g/day
**Fat (g)**	43 ± 21	37 ± 14	45 ± 22	0.280	**
**SFA (g)**	8.5 ± 4.5	7.2 ± 3.3	8.8 ± 4.7	0.253	**
**MUFA (g)**	8.4 ± 4.6	6.9 ± 3.6	8.8 ± 4.8	0.178	**
**PUFA (g)**	15.6 ± 9.6	13.5 ± 6.7	16.2 ± 10.2	0.378	**
**Cholesterol (mg)**	226 ± 153	177 ± 135	238 ± 156	0.196	<200 mg/day
**omega 6:omega 3**	10.4 ± 5.7	9.0 ± 5.3	10.7 ± 5.7	0.327	4:01
**Water (mL)**	1446 ± 639	1577 ± 664	1413 ± 635	0.411	750–1500 mL/day
**Vitamin A-IU**	1360 ± 2168	891 ± 1136	1478 ± 2530	0.386	700–900 IU
**Vitamin D-IU**	46 ± 39	30 ± 37	50 ± 39	0.110	600 IU
**Vitamin E (mg)**	2.3 ± 1.6	1.9 ± 0.8	2.4 ± 1.7	0.398	15 mg
**Vitamin K (µg)**	22 ± 78	18 ± 34	23 ± 86	0.825	90–120 µg
**Vit B1 (mg)**	0.7 ± 0.3	0.7 ± 0.2	0.8 ± 0.4	0.220	1.1–1.2 mg
**Vit B2 (mg)**	1.0 ± 1.7	0.7 ± 0.2	1.1 ± 1.9	0.395	1.1–1.3 mg
**Vit B3 (mg)**	13.8 ± 5.4	15.1 ± 5.3	13.5 ± 5.4	0.325	14–16 mg
**Vit B6 (mg)**	10.8 ± 28.0	8.3 ± 15.7	11.4 ± 30.4	0.729	13–17 mg
**Vit B12 (µg)**	1.8 ± 2.1	1.5 ± 1.1	1.9 ± 2.3	0.572	2.4 µg
**Biotin (µg)**	10.0 ± 9.8	9.7 ± 9.3	10.1 ± 10.0	0.907	30 µg
**Folate (µg)**	142 ± 189	96 ± 61	152 ± 209	0.399	1000 µg
**Vit C (mg)**	87 ± 73	64 ± 45	93 ± 78	0.209	75–90 mg/day
**Dietary Ca (mg)**	431 ± 262	311 ± 149	461 ± 276	0.063	<1000 mg
**Iron (mg)**	15 ± 14	11.8 ± 7.8	15.6 ± 15.1	0.377	**
**Dietary P (mg)**	872 ± 422	785 ± 212	894 ± 458	0.411	1000 mg
**Dietary K (mg)**	1475 ± 582	1407 ± 389	1492 ± 623	0.641	**
**Dietary Na (mg)**	2099 ± 918	1993 ± 732	2125 ± 962	0.647	<2400 mg
**Zinc (mg)**	8.0 ± 4.5	7.6 ± 3.1	8.1 ± 4.8	0.716	15 mg
**Magnesium (mg)**	231 ± 91	214 ± 58	235 ± 98	0.466	200–300 mg

The diet data are reported for the 65 acceptable reporters. Values are as Mean ± SD. DEI: Dietary energy intake, DPI: Dietary protein intake. SFA: Saturated fat, MUFA: Monounsaturated fat, PUFA: Poly unsaturated fat. IU: International Unit, Vit: vitamin, Vit E: Alpha tocopherol. BW: Body weight. Ca: Calcium, Na: Sodium, K: Potassium, P: Phosphorous. ** Individualized. ^a^ Mean values (between PEW and Non-PEW patients) sharing a common superscript were significantly different from each other using a one-way ANOVA (*p* < 0.05).

**Table 3:** Health and Nutrition assessments between PEW versus Non-PEW groups.

Assessments	PEW	Non-PEW	*p* Value
**MIS Score**	7.6 ± 3.1 (14) ^a^	5.3 ± 2.7 (65) ^a^	*0.004*
**Well-nourished < 5**	3.0 ± 0 (2)	3.1 ± 0.9(27)	0.911
**Malnourished ≥ 5**	8.4 ± 2.6(12) ^a^	7.0 ± 2.2 (38) ^a^	*0.030*
**ADAT Score**	3.0 ± 1.1 (10)	3.7 ± 1.6 (43)	0.159
**KD-QoL**			
**SF-12 Physical Health Composite**	43.7 ± 12.5 (15) ^a^	37.3 ± 10.4 (64) ^a^	*0.043*
**SF-12 Mental Health Composite**	50.1 ± 7.5 (15) ^a^	43.9 ± 9.6 (64) ^a^	*0.023*
**Burden of Kidney Disease**	35.4 ± 23.5 (15)	28.1 ± 26.9 (66)	0.337
**Effects of Kidney Disease**	72.6 ± 19.1 (13)	63.5 ± 16.2 (58)	0.082

Data were collected from the number of patients indicated in parentheses. Values are mean ± SD. ^a^ Mean values (between PEW and Non-PEW patients) sharing a common superscript were significantly different from each other using a one-way ANOVA MIS [27]: Malnutrition inflammation score. A score > 5 indicates malnourishment [20]. MIS has 10 components, each with four levels of severity, from 0 (normal) to 3 (very severe). The sum of all 10 MIS components ranges from 0 (normal) to 30 (severely malnourished); higher score reflects a more severe degree of malnutrition and inflammation ADAT [24]: Appetite and diet analysis tool. Scale: 1 = very good, 2 = good, 3 = fair, 4 = poor and 5 = very poor. KD-QoL [25] Subscale scores range from 0 to 100, with lower scores indicating poor self-reported QOL.

**Table 4:** Lipid profile and sub-fraction analyses: PEW versus Non-PEW patients.

	ALL (107)	PEW (20)	Non-PEW (87)
**TC (mg/dL)**	155 ± 40	135 ± 34 ^a^	160 ± 40 ^a^
**HDL-C (mg/dL)**	35 ± 11	38 ± 16	34 ± 10
**TG (mg/dL)**	178 ± 98	132 ± 51 ^a^	188 ± 103 ^a^
**LDL-C (mg/dL)**	85 ± 31	71 ± 29 ^a^	88 ± 31 ^a^
**Non-HDL-C**	121 ± 40	97 ± 28 ^a^	126 ± 41
**TG/HDL-C**	5.9 ± 4.0	4.2 ± 2.7 ^a^	6.4 ± 4.1 ^a^
**Large HDL (mg/dL)**	12.4 ± 8.1	16.6 ± 10.4 ^a^	11.4 ± 7.3 ^a^
**Small HDL (mg/dL)**	4.7 ± 2.4	3.4 ± 2.2 ^a^	5.0 ± 2.3 ^a^
**Mean LDL size (Å)**	267.9 ± 6.7	271.0 ± 3.4 ^a^	267 ± 7 ^a^
**LDL-Pattern, *n* (%)**			
***A***	63 (59%)	16 (80%)	47 (54%)
***B***	29 (27%)	2 (10%)	27 (31%)
***Intermediate***	15 (14%)	2 (10%)	13 (15%)

Data were analyzed for the number of patients indicated in parentheses. Values are Mean ± SD and n or %. TC: Total Cholesterol, HDL-C: High density lipoprotein, TG: Triacylglycerol/triglycerides, LDL-C: Low density lipoprotein, Type A: Athero-protective profile, Type B: Atherogenic profile, Intermediate: Has characteristics of both A and B. ^a^ Values sharing same superscripts between the groups were significantly different using one-way ANOVA (*p* < 0.05).

## Data Availability

Data available upon reasonable request.

## Supplementary Materials

The following are available online at https://www.mdpi.com/article/10.3390/nu14071469/s1, Table S1: Study Flow Chart. Table S2: Comparison between 2× and 3× weekly dialysis.
