# Supplementary material for: Protein Energy Wasting in a Cohort of Maintenance Hemodialysis Patients in Dhaka, Bangladesh
Source: Nutrients. 2022 Apr 1;14(7):1469. doi: 10.3390/nu14071469 (PMC9002623; doi:10.3390/nu14071469)
Supplement: Supplementary file 1 [file nutrients-14-01469-s001.zip › nutrients-1640833-supplementary.pdf]

Supplementary Table S1. Study Flow Chart

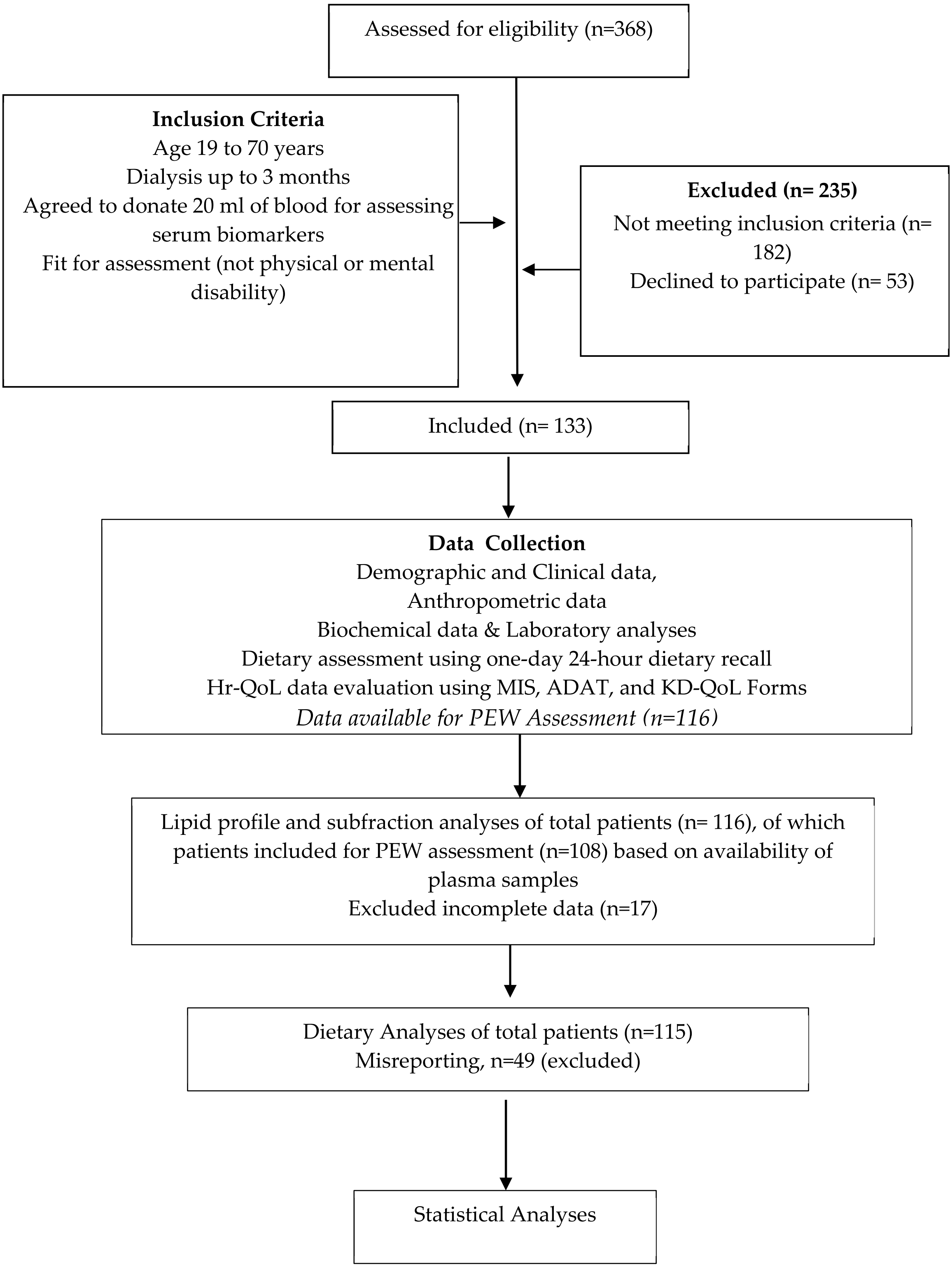

Supplementary Table S2: Comparison between 2X and 3X weekly dialysis

Characteristics	2X Weekly	3X Weekly	*P* value
**Age**	49±13 (81)	52±13 (48)	0.224
**Gender (M/F)**	36/45	25/23	
**Dialysis Vintage (months)**	25.5 ± 23.5[a] (77)	37.5 ± 24.4[a] (46)	0.008
**URR%**	67 ± 8[a] (50)	63 ± 9 [a] (40)	0.010
**Kt/V**	1.4±0.3 (30)	1.2±0.5 (27)	0.090
**Ferritin (ng/mL)**	376 ± 360 [a](38)	601 ± 476[a] (35)	0.025
**Albumin (g/dl)**	3.7±0.7 (60)	3.7±0.4 (44)	0.756

Values are mean±SD for the number of patients indicated in parentheses.
Urea Reduction Rate. Mean values sharing a common superscript were significantly different from each other using a one-way ANOVA($p<0.05$).

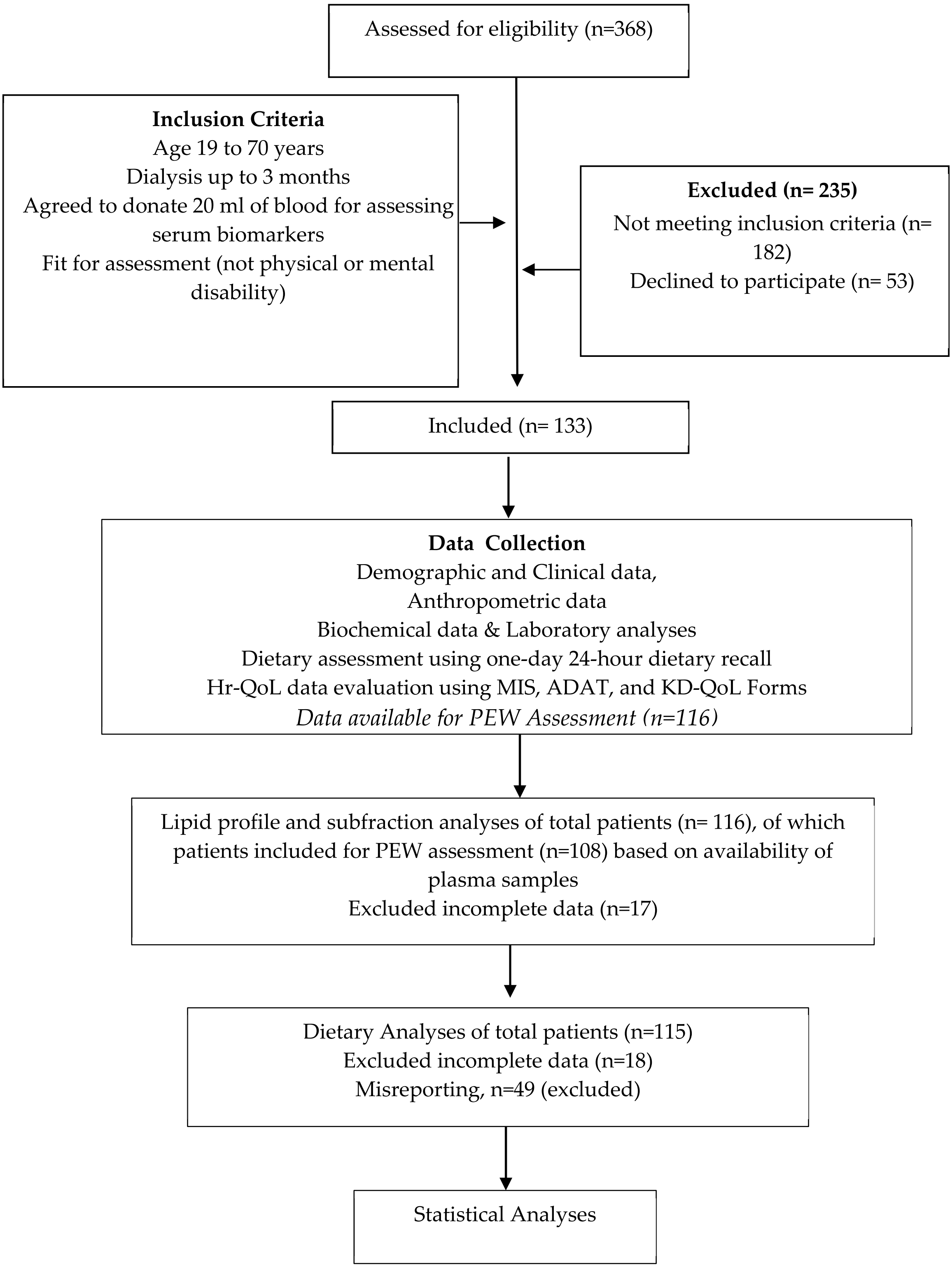
Assessed for eligibility (n=368)
Inclusion Criteria
Age 19 to 70 years
Dialysis up to 3 months
Agreed to donate 20 ml of blood for assessing serum biomarkers
Fit for assessment (not physical or mental disability)
Excluded (n= 235)
Not meeting inclusion criteria (n= 182)
Declined to participate (n= 53)
Included (n= 133)
Data Collection
Demographic and Clinical data,
Anthropometric data
Biochemical data & Laboratory analyses
Dietary assessment using one-day 24-hour dietary recall
Hr-QoL data evaluation using MIS, ADAT, and KD-QoL Forms
Data available for PEW Assessment (n=116)
Lipid profile and subfraction analyses of total patients (n= 116), of which patients included for PEW assessment (n=108) based on availability of plasma samples
Excluded incomplete data (n=17)
Dietary Analyses of total patients (n=115)
Excluded incomplete data (n=18)
Misreporting, n=49 (excluded)
Statistical Analyses
